# The Effect of Cognitive Behavioral Group Therapy on Children’s Self-Esteem

**DOI:** 10.3390/children8110958

**Published:** 2021-10-24

**Authors:** Naomi Epel, Ariela Abir Zohar, Adi Artom, Anne Marie Novak, Shahar Lev-Ari

**Affiliations:** 1 Shitot Institute, Ayelet Hashachar 6, Even Yehuda 4053079, Israel; arielazohar20@gmail.com (A.A.Z.); adiartom@gmail.com (A.A.); 2 Department of Health Promotion, School of Public Health, Faculty of Medicine, Tel Aviv University, Tel Aviv 69978, Israel; annemarien@mail.tau.ac.il; 3 Bob Shapell School of Social Work, Tel Aviv University, Tel Aviv 61390, Israel

**Keywords:** children’s self-esteem, cognitive behavioral group therapy, children’s cognitive behavioral therapy, CBGT, children’s group therapy, children’s wellbeing, children’s emotional resilience, school therapy, children’s therapy, therapeutic process, school teachers, CBT protocol

## Abstract

(1) Background: Self-esteem plays an important role in developing emotional resilience and wellbeing in children. Yet, there has been little related research on Cognitive Behavioral Group Therapy on this topic. Our aims were to assess the effect of the Child Self-Esteem CBT (CSE-CBT) protocol on children’s self-esteem in grades five and six; to assess the effect of the CSE-CBT protocol on the therapeutic process; and to explore the feasibility of delivering the CSE-CBT protocol in a school setting. (2) Methods: Eighty elementary school children in grades five and six, divided into four intervention and four control groups, attended 12 structured sessions using the CSE-CBT protocol, led by specially trained teachers. The children completed questionnaires to assess their self-esteem at the beginning and at the end of the study, and answered weekly questionnaires that assessed therapeutic process. Hierarchical linear modeling was used to analyze the data. (3) Results: The CSE-CBT protocol had a significant effect on improving children’s self-esteem over the course of the study, regardless of the children’s working alliance with the teacher leading the group. (4) Conclusions: The findings suggest that the CSE-CBT protocol has the potential to benefit children’s self-esteem and indicate that school teachers can be trained to administer the CBT-protocol.

## 1. Introduction

Self-esteem refers to the subjective perception of the self [1] and can be defined as a positive or negative attitude toward oneself [2]. It is an important variable in psychology research because of its significant correlation with emotional wellbeing [3,4,5,6,7,8]. Cultivation of self-esteem in early childhood reinforces positive values that can help children optimally cope with dynamic life situations [3,9,10,11,12,13], and is related to several markers of wellbeing, such as happiness, life satisfaction, self-confidence in social situations, sociability, and optimism [14,15]. However, low self-esteem among children is associated with negative thoughts, avoidance of new experiences, difficulty in managing conflicts [1,16], poor physical and mental health later in life [17], depression [18,19], loneliness [20], suicidal thoughts [21], suicide attempts [22], eating disorders [23,24], anxiety [25], behavioral problems [26], drug and alcohol addictions [27], as well as criminal and anti-social behavior [27]. It is not surprising, then, that high self-esteem has been identified as a buffer against the onset of mental illness in childhood [28] and it functions as a mechanism for reducing stress [29] even when they face extremely stressful situations [25,30]. Thus, developing self-esteem at a young age is crucial for achieving better ability to cope with difficulties, and for developing resilience and emotional wellbeing later in life [29,31]. 

The children who participated in the present study were in their early adolescence. Research has shown that in the 11–14 age group, social support has a greater effect on increasing or lowering self-esteem than does the child’s family, which had been the most influential factor until this point [32]. Therefore, at this age, treating self-esteem within the peer group could significantly contribute to the self-esteem of children [33] by enhancing their sense of belonging and ability to share and solve problems, enabling the peer group and the therapist to offer reinforcements and serve as role models. Group therapy can help children realize that some of the challenges they face are experienced by others as well, which contributes to alleviating the internal negative experience [34]. The group can simulate a “playground” where children practice the skills and interactions that they are likely to encounter in their daily lives [35,36,37]. Although research in this area is scarce, it indicates that group therapy for children to enhance self-esteem seems to be effective [38,39,40]. The most natural place for group therapy to improve children’s self-esteem is school [41], where children cope with stressful situations such as failure, isolation, rejection, or bullying [42]. This setting has been shown to be beneficial for improving self-esteem in group therapy based on the cognitive behavioral approach [34,43,44]. Modularized evidence-based practices are congruent with the goal of implementing effective care in school settings [45]. These programs address frequent barriers that are often seen in usual care settings, including high costs of services, waitlists, and transportation requirements [46], but require teachers trained to facilitate group sessions [46]. There is empirical evidence that school teachers can effectively lead Cognitive Behavioral Therapy (CBT) groups [47] and that students in such groups successfully comply with the CBT protocol, modify their thinking and behavior patterns, and acquire problem solving skills to the same extent as those who participate in groups led by professional therapists [48,49]. Studies suggest that this method may be used to make therapy more readily available in schools, enabling the benefit of a larger number of children [42]. Yet, most of the research on improving self-esteem with school-based therapy has analyzed interventions conducted by professionals. 

Cognitive behavioral group therapy (CBGT) is especially suitable for children in school settings because it is structured and goal-oriented [50]. One of the goals of CBT for coping with low self-esteem is to help individuals accept themselves as they are, explore their presumptions, undermine and weaken non-adaptive beliefs, and find beneficial alternatives [51,52]. CBT has proven highly effective in reducing mental and emotional distress [50,53,54,55,56] and in immediately alleviating distress in children [57]. It was shown to have long-term beneficial effects [53,54], helping to prevent future distress [58]. A meta-analysis showed that working directly on increasing self-esteem was more effective than prevention programs or programs designed to modify behavior or social skills [59]. The current study tested a direct protocol for improving self-esteem through group therapy with children, which, to the best of our knowledge, has not been adequately researched to date. Controlled studies on CBT for improving self-esteem in children have found this method to be highly effective [34,60,61,62]. The content of CBGT designed to increase self-esteem in children focuses on identifying the thinking patterns that produce negative thoughts, on modifying thinking strategies, and on problem solving [34]. The sessions follow a structured protocol that involves identifying thoughts, emotions, and behaviors, self-monitoring, cognitive construction, and exercises [55]. Each session is planned in advance and provides participants with the tools they need to change the problematic situation that brought them to the group using psycho-educational explanations, practicing new skills, and completing assignments at home [63]. The ability to learn and practice new tools and skills is supported by the group therapeutic process of a secure, supportive group of peers who encourage one another to share their experiences and process them with the help of the group [58]. The therapeutic processes (such as the therapeutic alliance between group members and the facilitator, the feelings of the participants in the session, and the meaning of the session for them) and their contribution to group therapy focused on increasing self-worth, have not been sufficiently researched.

Unlike research on adults, research on child and adolescent treatment has not clarified whether the youth–therapist alliance is a consistent predictor of outcome [64]. Studies have shown that the quality of alliance may not differ significantly between manualized and non-manualized treatment in practice settings [65,66]. Understanding the factors that may influence alliance in youth therapy, however, is an important research objective. Does self-esteem improve in children because of the therapy protocols used or because of variables related to the group therapy process that affects self-esteem, such as the child’s relationship with the group facilitator, or emotional experiences during the sessions? A study by Bosmans and Kerns addressed this issue and showed that the type of bond children form affects their ability to benefit from group CBT in general, and from the relationship with the facilitator in particular [67]. This is relevant in the case of CBGT, where the facilitator plays a dominant role throughout the lifecycle of the group [68].

The primary goal of this study was to assess the effect of the Child Self-Esteem CBT protocol (CSE-CBT protocol) on children’s self-esteem in grades five and six. The secondary goals of the study were: (1) To assess the effect of the CSE-CBT protocol on working alliance; (2) To assess the effect of the CSE-CBT protocol on children’s positive feelings and the meaning they attribute to the sessions; (3) To explore the feasibility of delivering the CSE-CBT protocol by schoolteachers that have been specifically trained for this type of intervention in a school setting. We hypothesized that an increase in children’s self-esteem will be greater in the intervention group than in the control group, and the stronger the therapeutic alliance (teacher and participants), positive feelings during the session, and positive meaning of the session indices correlates with changes to the participants’ self-esteem, especially in the intervention group. The outcomes of this study may help integrate self-esteem-enhancing interventions in schools as part of building children’s emotional resilience.

## 2. Materials and Methods

The study took place in a large elementary school in central Israel after it received the required approval from the Israeli Ministry of Education at 13 August 2017 (Permit No. 9599) and from the Human Subjects Review Committee of the Professional School of Psychology—Sacramento, CA on 14 November 2017. Although the study was submitted and approved by the ethics committees prior to data collection, no registration was made in a public trial registry. The socioeconomic status of students was in the average to low-average range, and most participants had two working parents. All children (n = 210) in the fifth and sixth grades were invited to participate in the study. The condition for participating in the study was the consent of the parents and the child to take part in it. Of all the children, 80 agreed to participate in the study and were randomly assigned to two groups: an intervention group and a control group. Each group was divided into four subgroups of ten children. Each subgroup contained both boys and girls, and students from both participating age groups. 

All the participants completed a Rosenberg Self-Esteem Scale (RSE) [2] questionnaire in the first and last sessions (sessions 1 and 12). At each session, they also completed the Working Alliance Inventory (WAI) [69] to assess the therapeutic relationship, and the Session Evaluation Questionnaire (SEQ) [70] to assess the children’s feelings about the session and its meaning for them. After each session, time was devoted to completing the questionnaires, which lasted about 15 min. The children received the questionnaires in the same order each time.

The intervention was based on a protocol that follows the cognitive behavioral approach, specifically, on the interventions developed by Epel [71]. As is common in CBT, the CSE-CBT protocol of each group session consisted of three parts: (a) Introduction to the topic and psycho-educational explanation. Each session introduced a new topic that helped children understand how the topic related to their personal and social lives; (b) Practice. The facilitator introduced new skills, practiced them with the children, and examined the influence of the skills on their thoughts, feelings, and behavior; (c) Home tasks. To implement the change, students were required to practice the skills learned during the session at home [71]. The outline of the sessions is described in Table 1. 

The sessions were facilitated by eight teachers who did not teach the participating children during the school year. They volunteered to stay after regular school hours for the purpose of this study. They received 15 h of training from the researcher, after which they were randomly divided into two groups. Four teachers led the intervention groups based on a 12-session (12-week) protocol for improving self-esteem, and the four other teachers led the group of children in the control groups and used the time for playing creative thinking games. The sessions were 45-minutes-long (the length of a school lesson) and were held on the same day and at same time each week, immediately after regular school hours ended.

The students in the intervention and control groups completed the Rosenberg Self-Esteem Scale (RSE) [2] questionnaire before and after the intervention process. RSE is considered to be the most commonly used and important questionnaire for assessment of self-esteem [75]. Its advantages are that it is short, clear, focused, and easy even for children to answer [76]. The questionnaire assesses the respondents’ self-esteem based on ten self-reported items. Respondents are instructed to select the answer that best represents them from four options: 0 = Completely disagree, 1 = Disagree, 2 = Agree, and 3 = Completely agree. An example of an RSE question is: “In general, I’m satisfied with myself.” Five of the scores are reversed before a final score is calculated by adding the scores for each of the items, for a final score ranging from 10 (minimum) to 30 (maximum). The questionnaire has been validated and found reliable [75,77]. The study by Blascovich and Tomaka [77] reported very good internal consistency (Cronbach’s α = 0.92). The questionnaire was administered to middle school students [78], and its reliability was found to be high (Cronbach’s α = 0.84). For the present study we used the Hebrew translation of the questionnaire [79] which was found to have high reliability (Cronbach’s α = 0.88).

The 36-item Working Alliance Inventory (WAI) [69] is based on the theoretical model described by Bordin [80], which was proved to be efficient for research of groups [81]. The questionnaire assesses three components related to the working alliance, namely, the quality of the patient–therapist bond, the degree of agreement on therapy goals, and the tasks assigned in the course of therapy. As one of the objectives of this study was to assess the bond between the group facilitator and the participants, as a function of changes in the participants’ self-esteem, we referred only to the first part of the questionnaire which includes 12 questions that relate directly to the relationship between the participants and the facilitator. The children and the facilitators completed the two versions (patient and therapist) of the first part of the questionnaire. The questionnaire contained 12 questions scored on a 7-point Likert scale, ranging from 1 = “Does not at all describe how I feel” to 7 = “Accurately describes how I feel.” Some of the questions were worded in the negative form, e.g., “I don’t feel comfortable with...” Higher scores indicated a better working alliance. Horvath and Greenberg reported good internal consistency for the therapist’s questionnaire (α = 0.93) and for the patient’s questionnaire (α = 0.87) [69]. The questionnaire had been translated into Hebrew and used in a previous study with children, showing good internal consistency for the group (α = 0.91) and the therapist (α = 0.88) [82]. For the present study, we also found good internal consistency of α = 0.84 for the patient (student) and α = 0.84 for the therapist (teacher). In the present study, the questionnaire was completed at the end of each session, to assess whether there was a change from the previous session in the relations between the facilitator and the group of peers from the perspectives of both the facilitator and the children.

The Session Evaluation Questionnaire (SEQ) [70] was tested on a sample of 218 participants during 2414 therapeutic sessions. Participants reported their assessment of the therapy based on three categories that were measured separately: (a) feelings during the session (e.g., “How happy or sad I am, confident or apprehensive, satisfied or angry”); (b) the meaning of the session for the participant (e.g., “Important or unimportant, special or ordinary”); and (c) feelings about the therapist (e.g., “Today I feel that my therapist was…”). As we used WAI to assess the quality of the mutual relations between the children and the facilitator, we skipped the third part of this questionnaire to avoid burdening the children. In the first two categories, the children were asked to circle the number that best reflected their feelings about the session and its meaning for them. Each category was presented in a six-point bipolar adjective format. The children were asked to describe how they felt during the session using one of the following pairs: confident-afraid, happy-sad, friendly-unfriendly, alert-drowsy, pleased-angry, excited-indifferent. Regarding the meaning of the session, the children were asked to describe how significant the session was using the following pairs of adjectives: good-bad, important-unimportant, easy-difficult, pleasant-unpleasant, special-ordinary. Higher scores indicate that the session was more significant. The study conducted by Stiles et al. reported an internal consistency of α = 0.80 for meaning and α = 0.92 for feelings [70]. In a study carried out in Israel with 93 children with learning disabilities from different schools, Freilich and Shechtman also used only the first two categories and reported an internal consistency of α = 0.66 for meaning and α = 0.87 for feelings [83]. In the present study, the questionnaire was completed at the end of each session to assess how the children felt during the session and the meaning of the session for them. Internal consistency during the session was α = 0.73 for feelings and α = 0.81 for meaning.

## 3. Results

**Figure 1:**
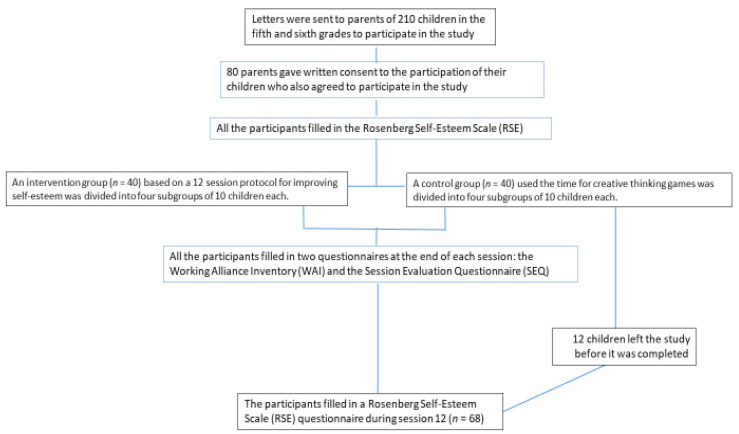
CONSORT diagram.

Eighty children (42 girls (52.5%) and 38 boys (47.5%)) were randomized to either the intervention or the control group. Study researcher (A.Z.A) generated the random allocation sequence using simple randomization (manual card shuffling tool) in a 1:1 ratio, enrolled the participants, and randomly assigned participants to either intervention or control group. Each group was divided randomly (simple randomization) to four subgroups of ten children. Twelve children from the control group (30%) left the study before it was completed because of extra lessons offered by the school in core subjects (English, math, and language studies) during the same time slot as the therapy sessions. The other 68 children were in all 12 sessions, and there was no dropout from the intervention group. The diagram of the research process in accordance with CONSORT guidelines is shown in Figure 1.

We used Little’s MCAR test [84] to assess the missing values. The results showed that the missing data were missing entirely at random (χ^2^(1251) = 1268.40, *p* = 0.36). To handle the missing data, we used multiple imputations [85] on the sample of 68 participants, in both the intervention and control groups. As findings may be affected by the composition of each group, its facilitator, or both, as well as by the intervention program, we checked whether the groups could influence the results using a series of interclass correlation (ICC) analyses to assess the internal consistency of each group, for each of the research indices. 

The statistical analyses selected to assess our hypotheses were based on hierarchical linear models [86] which support multilevel nested data. In the current study, which involves estimations of change over time, we used a series of HLM-3 models to handle three levels of nested data: repeated measurements, participant, and group. Results of the ICC analysis indicated that the perception of the working alliance from the perspectives of the teacher and the students, as well as the meaning of the session had good internal consistency within the groups, as shown in Table 2. Furthermore, the children’s working alliance indices showed that positive feelings during the session and the Meaning of the session contributed significantly to explaining the variability of the indices for the group. Means and standard deviations of all measures for all groups (intervention/control) at baseline and Study completion are presented in Table 3. 

### 3.1. Assesment of Changes in Self-Esteem Following the Intervention

We assessed our hypothesis that the increase in students’ self-esteem would be greater in the intervention group than in the control group. We measured self-esteem at two points in time: before the intervention program began (T1) and after the final session (T12). We conducted an HLM to this end at the repeated measurements level. We incorporated a predictor variable that characterizes the time matrix and coded it using 0 and 1 to capture the difference between the two points of measurement. At the participant level, we incorporated the group affiliation variable as the predictor (0 = control group; 1 = intervention group) and tested the interaction between group affiliation and time matrix. This enabled us to assess whether the change in the level of self-esteem over time was dependent on the group. To clarify the meaning of the significant interactions, we used the procedure described by Preacher, Curran, and Bauer [87], based on analysis of simple slopes and adapted for the HLM environment. We did not incorporate any predictors at the group level, but this level enabled us to assess the research hypothesis while considering the contribution of the group to the explained variance of the degree of change in self-esteem over the course of the study.

**Figure 2:**
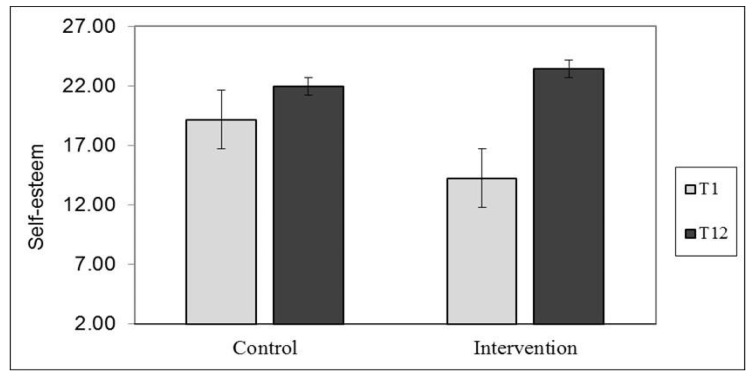
Changes in self-esteem from T1 to T12, as a function of group.

The model indicated that self-esteem increased significantly between T1 and T12, b = 6.00, SE = 1.02, t = 5.88, *p* < 0.001, and that the increase was dependent on the group (i.e., a significant interaction was found between time matrix and Group), b = 6.42, SE = 2.04, t = 3.14, *p* = 0.002 (Figure 2). A simple slope analysis showed that, as hypothesized, there was no significant change in self-esteem in the control group over the course of the study, whereas self-esteem increased significantly in the intervention group during this period, as shown in Figure 2.

### 3.2. Therapeutic Process and Self-Esteem

We examined whether changes in the working alliance (teacher and participants), positive feelings, and meaning of the session indices correlated with changes to the participants’ self-esteem as a function of group. We used a moderation model in which the correlation between changes in the working alliance (teacher and student), positive feelings, and meaning of session indices on one hand, and changes in self-esteem on the other is group-dependent, that is, differs between the intervention and control groups. To explore this possibility, we conducted another series of HLMs. For the repeated measurements level, we used the time matrix variable as a predictor and coded it 0 and 1 to capture the change between the two measurement points. This level also included the degree of change in the working alliance, feeling, and positive meaning indices (using a separate model for each of these indices because of the limited sample size) as predictors, and the interaction of these indices with the time matrix variable. At the participant level, we used the group affiliation variable as the predictor (0 = control group; 1 = intervention group), as well as the interaction between the group affiliation and time matrix variables, and the working alliance, feeling, and positive meaning variables. This made possible the assessment of whether the correlation between working alliance, positive feeling, and meaning of session depends on the group, on measurement time, or both. We did not use any predictors at the group level. The HLM coefficients and their significance are shown in Table 4.

**Figure 3:**
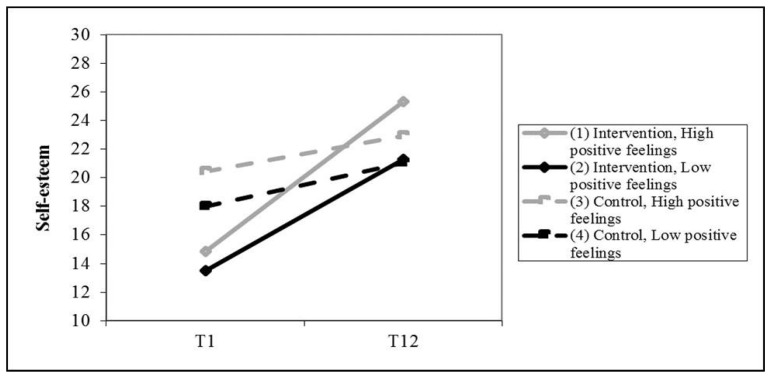
Correlation between positive feelings during the session, and change in self-esteem as a function of group.

Finally, we found that the degree of change in self-esteem depended on the participants’ positive feelings during the session and on the group (a significant three-way interaction between group, measurement time, and positive feelings during the session). As shown in Figure 3, the self-esteem of participants in the intervention group improved significantly between T1 and T12, but it remained unchanged in the control group. Yet, the improvement in self-esteem was greater for participants with more positive feelings during the sessions (one standard deviation about the average positive feelings score in the sample) than for those who displayed less positive feelings during the sessions (one standard deviation below average for the sample). The other effects were not statistically significant, as shown in Figure 3. HLM coefficients and their significance for assessing correlations between the Working alliance, Positive feelings, and Meaning of the session indices, and the change to self-esteem as a function of group are shown in Table 4.

## 4. Discussion

The results of the present study demonstrate that the CSE-CBT protocol is a beneficial tool for increasing children’s self-esteem in grades five and six in a school setting. The improvement in self-esteem was greater for participants with more positive feelings during the sessions. In the intervention group, children’s self-esteem improved over the course of the study regardless of their working alliance with the teacher leading the group. Finally, our study suggests the possibility that schoolteachers can be trained to administer a customized CBT protocol in a school setting to improve children’s self-esteem. 

We have demonstrated that there was a significant increase in the self-esteem of children in grades five and six after participating in the CSE-CBT protocol group. These findings are consistent with previous studies involving elementary school children [34,58,60] which also indicated that intervention programs based on the cognitive behavioral approach were effective in improving self-esteem in children. CBT-based intervention programs have been found to be particularly effective for children in the 10–12 age group [46,73], enabling children to comprehend the connection between thoughts, emotions, and behaviors, and to develop better control over them [88]. The present study suggests that methodical use of the protocol can help children improve their self-esteem, acquire tools to identify their thoughts, emotions, and behaviors, and to learn strategies for changing them. The self-esteem of children in the intervention group improved significantly between the first and last sessions, compared to that of children in the control group. 

In general, we found a positive correlation between the working alliance reported by the children and the group facilitator in both the intervention and control groups, without a significant difference between the groups. Over the course of the study, the working alliance in all groups became stronger from the perspectives of both facilitators and participants. The children’s self-esteem in the intervention groups improved over the course of the study regardless of their working alliance with the teacher leading the group. We found that the intervention group did not benefit from a strong working alliance. At the same time, however, this attests to the power of the intervention program, which improved self-esteem regardless of the ability to create a working alliance. Despite an extensive search for studies in this area, we were unable to find an adequate explanation for how a therapeutic protocol can be more effective than the relationship with the facilitator of the therapeutic group. A possible direction for the findings obtained in the present study is obtained from a recent study that examined relationships between various aspects of therapeutic alliance with treatment outcome, adherence, and attrition in Internet-delivered cognitive behavioral therapy (ICBT) for panic disorder with a newly developed self-report alliance measure [89]. Results indicate that the patient experience of the alliance in ICBT includes two aspects, each uniquely contributing to the outcome; patient connection to the program is related to the symptom outcome, whereas the dyadic relationship with the therapist serves as the glue to allow the treatment to hold. A study that assessed different types of therapy, including protocol-oriented therapy, found that the use of manuals was related to a stronger youth–therapist alliance in the early phase of treatment [65]. This means that the working alliance is not necessarily dependent on a particular type of therapy, but rather is a function of personality and environmental factors [65]. In addition, the protocol included exercises used during the sessions to help participants fully acquire the new skills. Exercises performed at home helped internalize these skills and provided additional opportunities for practicing the content covered in the session [71,90,91,92,93,94,95].

Another finding that applies to both the intervention and control groups was related to the positive feelings and the meaning that the children attributed to the sessions throughout the study, without a significant difference between groups. The positive feelings, however, improved self-esteem only in the intervention group, in correlation with the participant’s positive feelings during the sessions. The findings also indicate that the more positive the feelings during the session, the more positive the meaning was attributed to the session in the intervention group. Therefore, as hypothesized, the improved self-esteem of children in the intervention group interacted significantly with their positive feelings, compared to self-esteem in the control group, which remained almost completely unchanged over the course of the study. Contrary to an earlier study [96], in which the SEQ assessed positive feelings and meaning of the sessions for the participants and did not find differences between groups in which different protocols were used, in the present study the tool was sufficiently sensitive to detect changes that occurred during the study in both the intervention and control groups.

The correlation we found between positive feelings and increased self-esteem is reinforced by Landreth, who showed that group therapy contributes to an increase in positive feelings toward oneself through learning about themselves and about other children [36]. Working in a group setting creates a “playground” for children, giving them an opportunity to develop important social skills such as sharing, conflict resolution, receiving and giving assistance, decision making, delaying gratification, and more [35,36,37]. The act of sharing experiences with a group can help children enhance the quality of their relationships and contribute to improving their self-esteem [97]. Groups of this kind can contribute to enhancing self-esteem as a result of the mutual feedback, the emotional attachment, and the closeness between the group members.

The findings in the present study suggest the possibility that teachers can manage effectively therapeutic protocols designed to enhance self-esteem in children, if professionally trained as facilitators [46]. Teachers who receive appropriate training can also learn to incorporate the new skills they acquire in their regular classrooms which might affect the self-esteem of students who do not participate in the group intervention programs. Further research is needed to assess this possibility.

The present study has several limitations. One has to do with the children’s social desirability when completing forms, especially on a weekly basis. We tried to overcome this limitation by allowing participants to answer the questionnaires anonymously, using numbers rather than names. A second limitation is the difference in self-esteem levels of the study groups at baseline. The participants that had been randomized to the control group had higher self-esteem levels at baseline than those randomized to the intervention group. We used HLM analysis comparing differences in change over time (and not end-point values) to mitigate this limitation. Finally, implementing interventions in schools, and particularly to recruiting students, because of the need to remain longer at the school. We overcame this difficulty by sending regular reminders to students, and continually encouraging them to attend the sessions. Positive reinforcements (refreshments, small prizes) for the children who remained after their schooldays for the study, both in the intervention and in the control groups, also helped mitigate this limitation.

Although the results of the present study suggest that the CSE-CBT protocol has the potential to benefit children’s self-esteem in a school setting, more research is required to confirm these findings and contribute to evidence-based practice. It is especially important to confirm the effect of the CSE-CBT protocol in a larger population, with a larger sample, and with different age groups.

## Figures and Tables

**Table 1:** Outline of the sessions.

Meeting Number	The Subject	Purpose of Meeting
1	Introduction and introductory games	Create a safe place for the children where they can meet with the facilitator and other participants.
2	Identity card creation	Who am I, what is special about me, and how do I speak about myself and my environment.
3	What do I feel	Identify, understand, and express feelings and emotions.
4	What causes a certain emotion to rise	Teach children what automatic thinking is and how it affects us, with the aim of enhancing the experience of controlling our internal processes.
5	Thoughts, emotions, and behavior	Understand the connection between thoughts, emotions, and behavior through the game “Bouncing Thoughts” [71]
6	Creating de-fusion	Create a distance between thought and reality through refutation processes. Using the book *Tigger Tigger Is it true?* [72]
7	My self-esteem	Identify and normalize situations that reduce self-esteem.
8	The “Secret Jar”	Acquire open self-control skills [73], to change self-talk about events with potential for reducing self-esteem. Using the book: *Grandfather Jamiko’s Secret Jar* [74]
9	The “Magic Shield”	Create resilience against complex social situations, using closed self-control skills [73]. Using the book *Grandfather Jamiko’s Secret Jar* [74]
10	Conflict management	Learn effective verbal expression in various social situations, and practice “the words we use” [71].
11	The “self-love box”	Preparing a special box for self-strengthening and empowerment of self-esteem.
12	Termination and separation	Sharing the process that participants went through during the sessions using therapeutic cards.

**Table 2:** ICC test results and the significance of the variance associated with the groups for the main research indices.

			Variance Associated with the Group Distribution		
	ICC_(1)_	ICC_(K)_	χ^2^	*df*	*p*-Value
Self-esteem	56.	11.	12.95	7	073.
Working alliance (teachers)	**71.**	04.	4.59	7	710.
Working alliance (students)	**94.**	21.	**127.05**	7	001.
Positive Feelings during the session	36.	01.	**15.22**	7	033.
Meaning of the session	42.	01.	**21.51**	7	003.

Note: ICC (1) reflect within class agreement in scores; ICC(k) variance accounted by differences between classes. Significant values appear in bold.

**Table 3:** Means and standard deviations of all measures for all groups (intervention/control) at baseline and Study completion.

Characteristic	Control, N = 28 ^1^	Intervention, N = 40 ^1^	*p*-Value ^2^
Self-Esteem (SE)—Baseline	19.1 (5.8)	14.2 (5.2)	<0.001
SE—Study completion (Week 12)	21.7 (6.5)	23.9 (5.2)	0.16
Working alliance teachers (WAT)—Baseline	5.33 (1.05)	5.65 (1.09)	0.23
WAT—Study completion (Week 12)	6.50 (1.01)	6.27 (0.81)	0.32
Working alliance students (WAS)—Baseline	5.37 (0.62)	5.86 (0.91)	0.010
WAS—Study completion (Week 12)	5.93 (0.63)	6.44 (0.24)	<0.001
Positive Feeling—Baseline	5.35 (0.80)	5.43 (0.65)	0.63
Positive Feeling—Study completion (Week 12)	5.86 (0.55)	5.92 (0.20)	0.62
Meaning of the session—Baseline	5.25 (0.96)	5.57 (0.57)	0.13
Meaning of the session—Study completion (Week 12)	5.99 (0.06)	5.98 (0.07)	0.55

^1^ Mean (SD), ^2^ Welch Two-Sample *t*-test.

**Table 4:** HLM coefficients and their significance for assessing correlations between the working alliance, positive feelings, and meaning of the session indices, and the change in self-esteem as a function of group.

		Self-Esteem		
		*B*	SE	*t*
Repeated measurements level	Time	5.98 ***	77.	7.76
	Group × Time	7.01 ***	1.54	4.55
	Working alliance (teacher)	66.	41.	1.63
	Group × Working alliance	2.14 **	81.	2.63
	Time × Working alliance	1.00-	1.21	83.-
	Group × Time × Working alliance	48.-	2.41	20.-
Participant level	Group	2.19-	1.71	1.28-
				
Repeated measurements level	Time	6.09 ***	80.	7.61
	Group × Time	6.30 ***	1.60	3.92
	Working alliance (students)	28.-	48.	58.-
	Group × Working alliance	51.	96.	53.
	Time × Working alliance	20.-	92.	21.-
	Group × Time × Working alliance	1.19-	1.85	64.-
Participant level	Group	1.70-	1.39	1.22-
				
Repeated measurements level	Time	5.95 ***	90.	6.61
	Group × Time	6.37 ***	1.80	3.54
	Positive feeling	1.21 ***	33.	3.68
	Group × Positive feeling	26.	66.	39.
	Time × Positive feeling	54.	36.	1.50
	Group × Time × Positive feeling	1.61 *	72.	2.24
Participant level	Group	1.86-	1.39	1.34-
				
Repeated measurements level	Time	6.09 ***	82.	7.47
	Group × Time	6.65 ***	1.63	4.08
	Meaning of the session	28.	53.	53.
	Group × Meaning of the session	73.	1.06	69.
	Time × Meaning of the session	1.09-	93.	1.18-
	Group × Time × Meaning of the session	47.-	1.85	26.-
Participant level	Group	1.89-	1.41	1.34-

* *p* < 0.05. ** *p* < 0.01. *** *p* < 0.001.

## Data Availability

Data might be available upon request from the corresponding author (N.E.), and according to the guidelines of Israeli Ministry of Education.
